# Kinetic study of the metal-dipeptide complex with ninhydrin facilitated by gemini (m-s-m) surfactant micelles

**DOI:** 10.1038/s41598-020-61001-6

**Published:** 2020-03-05

**Authors:** Naved Azum, Dileep Kumar

**Affiliations:** 1 Chemistry Department, Faculty of Science, King Abdulaziz University, Jeddah, 21589 Saudi Arabia; 2 Division of Computational Physics, Institute for Computational Science, Ton Duc Thang University, Ho Chi Minh City, Vietnam; 3 Faculty of Applied Sciences, Ton Duc Thang University, Ho Chi Minh City, Vietnam

**Keywords:** Physical chemistry, Chemistry

## Abstract

The three Gemini (m-s-m; m (head group) = 16 and s (spacer) = 4, 5, 6) surfactants have been synthesized and their impact on reaction of zinc(II)-glycylleucine complex ([Zn(II)-Gly-Leu]^+^) and ninhydrin were studied at temperature (343 K) and pH (5.0) using spectroscopic method. Influence of several factors, viz., [Zn(II)-Gly-Leu]^+^, [ninhydrin], temperature and pH were also carried out on title reaction in geminis. Rates of reaction are the first-order path in concentration of [Zn(II)-Gly-Leu]^+^ complex and fractional order path in concentration of ninhydrin. The catalysis of gemini 16-s-16 surfactant micelles was investigated below and above their critical micelle concentration (cmc) value and detailed elaboration were provided in the text. In the present case, rate constants, k_ψ_, increased on increasing geminis ([gemini] are below their cmc, region I) and stayed nearly constant (region II). The shape of (region I and II) surfactants ([gemini] = 0 to 400 × 10^−5^ mol dm^−3^) are similar to a cetyltrimethylammonium bromide, CTAB (single hydrophilic head group and hydrophobic part). Later, a sharp increment in rate was observed with higher [gemini] (region III, (Fig. 5). The study was catalyzed and accelerated quite enough by geminis (at concentrations below their cmc) compared to aqueous. An appropriate mechanism has been proposed for accounting for the distribution of reactants between aqueous and micellar pseudo phases. Resulting kinetic data were used to determine the binding constants of micelle-substrate (K_B_) and micelle-ninhydrin (K_Nin_).

## Introduction

Micellar study on the reaction rate is a key phenomenon for researchers and scientists due to analogies between their reaction with biological activities^1,2^. Knowledge of surfactant micelle behavior on a biological system is extremely significant as the binding of naturally occurring substrates or added surfactant micelle may affect the biological process. Similarly, the existence of surfactant molecules and substrate molecules may account for the micellar study. In micellar media, reaction rates were influenced by electrostatic and hydrophobic interactions. It depends upon the extent of incorporation/association between substrate and surfactant aggregates^3–8^.

Surfactants have several applications in different fields (fundamental as well as applied)^9^,^10^. Gemini surfactants have a keen interest due to consist of two mono cationic moieties (hydrophilic head groups) and two long alkyl chain (hydrophobic group) attached by a flexible or rigid spacer chain length near or close to head groups. Besides lowering surface tension, gemini surfactants have some exceptional properties which include low cmc value, high surface activity, etc.^11,12^.

Furthermore, geminis exhibit advanced aggregation features and unusual wetting power capacity^13–15^. Due to their distinctive quality, they are employed for several purposes, such as household things, cleaning, pharmacy, cosmetics, gene therapy, in micellar catalysis and so on^16–21^.

Ninhydrin reactions are one of the most fundamental and biochemical studies. Ninhydrin, a chemical reagent, is used enormously for detection of amine functional group wherein it reacts with an amino group and generates diketohydrindylidene-diketohydridamine (DYDA)^22–30^. The color of the product formed between the reaction of ninhydrin and amino acids disappears at room temperature, several efforts were performed towards the stability of products that include the formation of the metal complex with amino acids, change of solvent, presence of conventional monomeric surfactant^28,31–33^. However, studies on ninhydrin with peptide are very scanty^34–37^. Reaction on ninhydrin with metal-peptides has also been performed in surfactant medium towards the increment of yield; hence, enhanced sensitivity. But, the study needs to be developing more for advanced outcome^3^.

In the recent era, there has been growing keen attention in protein due to their several uses in biosciences, foods, biotechnology, etc.^38,39^. Peptides, viz., oxytocin, vasopressin, luteinizing hormone-releasing hormone (LHRH) and opioid serve as a vital role in biological systems. These peptides are susceptible to enzymes. Proteins/peptides are an important class of compounds that play as antigen-presenting elements for the cell-mediated immune system. They have also different functions which include metabolic process, information transfer, and pharmaceuticals. Peptides act as building blocks in protein synthesis and consist of more difficult structural arrangements and constituents of protein as compared to amino acids. So, a kinetic study of the metal-dipeptide complex with ninhydrin facilitated by gemini (16-s-16) surfactant micelles is of great interest.

A number of articles have been published on the surface activity of gemini surfactants and their morphologies and it has been found that gemini surfactant is advanced in properties and applications than conventional monomeric surfactant (a similar single hydrophilic head group and hydrophobic part)^40–42^. But, the influence of gemini surfactants on rates has not attracted due attention. Therefore, we have synthesized and characterized three dicationic gemini surfactants, quaternary ammonium salts, (m-s-m type; m = 16 and s = 4, 5, 6) and their influence on the reaction rate of [Zn(II)-Gly-Leu]^+^ with ninhydrin has been investigated.

## Experimental

### Materials

Chemicals employed throughout the study were CH_3_COONa (99%, Merck, India), CH_3_COOH (99%, Merck, India), ninhydrin (99%, Merck, India), glycylleucine (99%, Loba Chemie, India) and zinc sulfate heptahydrate (99%, Merck, India). All of the above chemicals were used without any further surplus purification. For synthesizing gemini surfactants employed chemicals were 1,6-dibromohexane (>97%), 1,5-dibromopentane (>98%), 1,4-dibromobutane (>98%) and N,N-dimethylhexadecylamine (95.0%). These chemicals were purchased from Fluka, Germany. Other chemicals used in the current experiments were of AR grade. The specific conductivity of water employed during the whole study was (1–2) × 10^−6^ ohm^−1^ cm^−1^. Stock solutions of reactants and surfactants were prepared by dissolving requisite amounts in CH_3_COONa-CH_3_COOH buffer solution (pH 5.0). The buffer of pH 5.0 prepared by mixing of 30 cm^3^ of 200 mmol.kg^−1^ CH_3_COOH and 70 cm^3^ of 200 mmol.kg^−1^ CH_3_COONa^43^. The solutions were made freshly as per the necessities. To note the pH of the solutions, measurements were carried out on pH meter (ELICO LI-122, Hyderabad, India). In respect to achieving the composition of reaction products produced on the title reaction, Job’s method was applied in gemini surfactant media. It was identified that both the reactants (each mole of ninhydrin and [Zn(II)-Gly-Leu]^+^) associated to form the product.

#### Synthesis of cationic surfactants (m-s-m type gemini)

**Scheme 1:**
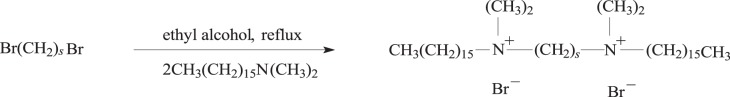
Synthetic route and structure of geminis. Where s = 4, 5, 6.

As shown in Scheme 1, cationic surfactants (m-s-m type gemini) were synthesized and characterized by the following steps.

*α*,*ω*-dibromoalkane (*s* = 4, 5, 6) and N, N-dimethylcetylamine were mixed in dry ethyl alcohol solvent into a 2 L flask and their molar ratio were kept at 1:2.1. The mixture was refluxed and stirred at temperature (353 K) for two days.

Progress of mixed system was checked and monitored by thin-layer chromatography, TLC, at regular time intervals. The solvent was removed under vacuum pressure; consequently, the crude product was obtained. This crude was recrystallized by the mixed solvent of ethyl acetate and ethyl alcohol. Thus, we achieved pure gemini surfactants. The purity of synthesized cationic surfactants (gemini) was ascertained by ^1^H NMR and C, H, N analysis^44,45^.

## Methods

### Surface tension measurements for cmc determination

**Figure 1:**
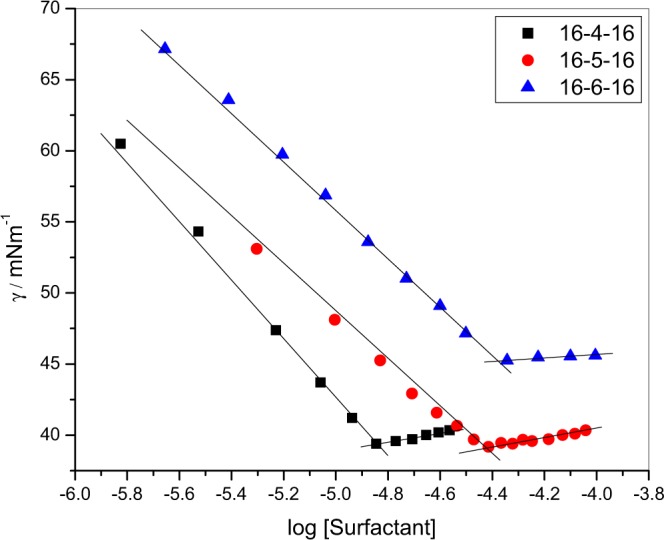
Plots of surface tension (γ) with log [surfactant].

The surface tension measurements were performed to determine the cmc and surface parameters of gemini surfactants. The attention tensiometer (Sigma 701, Germany) with a platinum ring was used for these experiments. The ring was cleaned on an ethanol flame before each experiment. To maintain the experimental temperature (±2 K) a thermostat was used. The values of surface tension decrease continuously and at a point and become constant (Fig. 1). The constancy in surface tension vs. concentration graph is taken as cmc.

### Conductometric method for cmc determination

**Figure 2:**
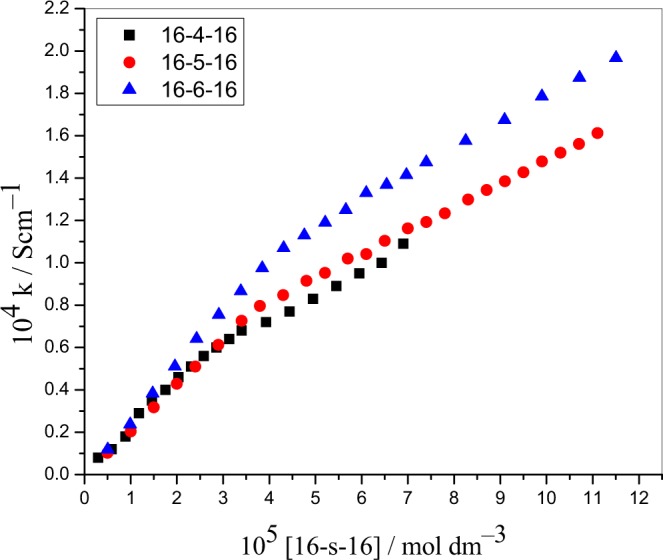
Representative plots of specific conductivity (κ) vs 16–s–16 gemini surfactants (mol dm^−3^) in an aqueous medium.

The conductometric method was also used for determining the cmc values of gemini surfactants by using an Equiptronic conductivity meter (EQ 661, India) with cell constant 1.0 cm^−1^. The material of the electrode used was made up of a PVC sleeved electrode. [ninhydrin] (=6 × 10^−3^ mol dm^−3^) and [Zn(II)-Gly-Leu]^+^ (=2 × 10^−4^ mol dm^−3^) were used for the cmc measurements. After each addition, the conductivity of the resultant solution was noted, thoroughly, to mix and attain equilibrium at reaction temperature using a flowing water bath. All studies were performed at least in triplicate. The plots between specific conductance and concentration showing the intersection between two straight lines were used to get the cmc values of gemini surfactants (Fig. 2)^46,47^. During the whole set of experiments, cmc values of gemini surfactants determined are listed in Table 1.

**Table 1:** Critical micelle concentration (cmc) values along with surface parameters determining by the surface tension and conductivity measurements.

Parameters	16-4-16	16-5-16	16-6-16
Tensiometry			
10^5^ cmc (mol dm^−3^)	1.458	3.802	4.365
p*C*20	5.340	5.120	4.660
10^6^*Γ*_max_ (mol m^−2^)	1.010	0.790	1.070
*A*_min_ (nm)	1.650	2.100	1.550
*Π*_cmc_ (mNm^−1^)	30.66	30.81	24.92
**Conductometry**			
10^5^ cmc (mol dm^−3^)	1.930	3.821	4.420
g	0.512	0.478	0.530
**Conductometry (water and complex with ninhydrin)**			
10^5^ cmc (mol dm^−3^)	3.40	4.20	4.40

### Kinetic study

The reaction of ninhydrin and complex was performed by UV-vis spectrophotometric technique (Kyoto, Japan). Solution mixture (zinc sulfate heptahydrate, glycylleucine (Gly-Leu), gemini surfactant and buffer with required quantities) was positioned in a reaction vessel. The vessel was left for equilibration in a water/oil bath at the reaction temperature. After that, the desired amounts of ninhydrin solution (placed separately in the same bath) were transferred to the vessel and the reaction was started. The absorbance was recorded by observing the formation of a colored-product at regular time intervals at *λ*_max_ = 310 nm. Each measurement has been made in triplicate.

### Calculation of rate constant (k_ψ_)

Whole calculations were computed by using a linear least-squares regression technique. The value of the reported rate constant was given as an average of duplicate runs. Observed k_ψ_-values were reproducible under the set of the present study. Rest info on the kinetic method is focused somewhere in the literature^31^.

### Spectra

**Figure 3:**
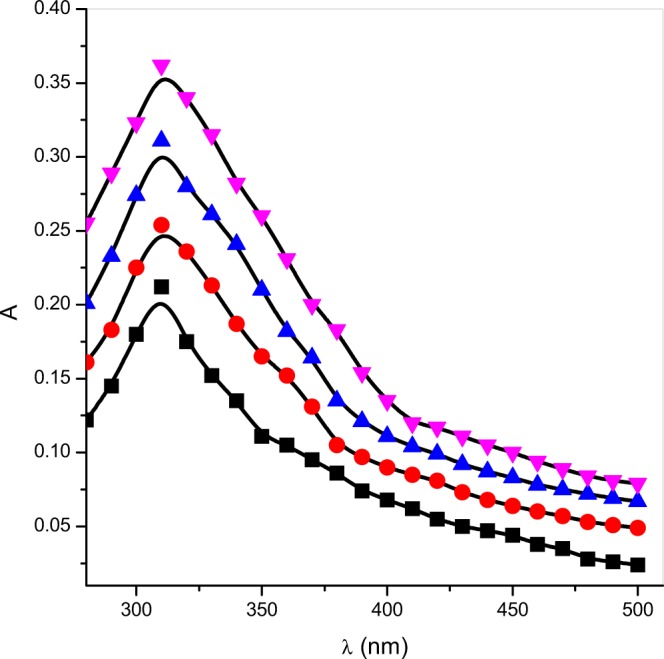
Plots of absorbance A vs. *λ* for ninhydrin and [Zn(II)-Gly-Leu]^+^ reaction in aqueous and gemini micellar media: (■) aqueous, (●) 16-6-16, (▲) 16-5-16, (▼)16-4-16. *Experimental conditions*: [ninhydrin] 6 × 10^−3^ mol dm^−3^, [Zn(II)-Gly-Leu]^+^ = 2 × 10−4 mol dm^−3^, [16-*s*-16] = 30 × 10^−5^ moldm^−3^, temperature = 343 K and pH = 5.0.

The absorbance of the product on the study of ninhydrin and [Zn(II)-Gly-Leu]^2+^ complex are noted at end of the reaction and shown at temperature (343 K) and pH (5.0) as UV-vis spectra in Fig. 3. Figure 3 confirmed that absorption maxima were observed the same at *λ*_max_ (=310 nm) in both the media. No change in *λ*_max_ in aqueous and micellar media concluded that the product formation was the same in both the systems.

## Results

### Micellization and surface properties

At low concentration, the aqueous solution of surfactants behave like electrolytes solution and monomers are found in the free state. However, at a specific concentration (cmc) the monomers tend to aggregates and form micelle. In the homologous series the cmc values logarithmically decrease with the number of carbon atoms in the chain (N_C_) as follow the relation:

1 $$\log ({\rm{cmc}})={\rm{A}}-{{\rm{BN}}}_{{\rm{c}}}$$

where A and B are constants. The hydrophobic interaction is the major driving force for micelle formation. During micelle formation water molecules in the hydration shells around the hydrophobic chain are released and entropy increases. With the increase of hydrophobic chains, more water molecules are release and micellization at lower concentration take place. The increase in the length of the chain by one –CH_2_ group decreases the cmc by 50%. The gemini surfactants have two hydrophobic chains so the tendency to form micelle is more than conventional surfactants. Hence the cmc of 16-s-16 is about 27% less than that of its predecessor CTAB.

The cmc values of gemini surfactants by surface tension and conductance measurements are given in Table 1 and are in good agreement with the reported values in literature^45^.

For the determination of the cmc of amphiphiles, the surface tension is the most acceptable technique. The cmc value can be examined by the plot of surface tension vs. amphiphile molar concentration (Fig. 1). One can see from Fig. 1 that the surface tension decreases continually up to a certain point, after that it almost constant, which means surface tension has achieved at saturation. The breakpoint or turning point in the graph specifies the formation of the micelle or cmc. To better show the limit of the surfactant to diminish the surface tension of the solution, π_*cmc*_, namely the effectiveness of the surface tension diminishment is presented here. π_*cmc*_ is the surface pressure at the *cmc* and is characterized as follows

2 $${\pi }_{cmc}={\gamma }_{o}-{\gamma }_{cmc}$$

where *γ*_*o*_ and *γ*_*cmc*_ are the values of surface tension of water and surfactant solution at the *cmc* respectively. The *π*_*cmc*_ are listed in Table 1. It is clear from the table that the 16-6-16 has a lower value of *π*_*cmc*_. The surface activity (pC20) of surfactant molecules has a special role in industrial applications. It may define as the surfactant concentration at which the surface tension value reduces by 20 mNm^−1^. Among the three gemini surfactants currently studied 16-4-16 is more surface-active (Table 1). The Gibbs equation was used to determine the surface excess (Γ_max_) at the interface as^48^

3 $${\Gamma }_{max}=-\frac{1}{2.303nRT}{\mathrm{lim}}_{C\to cmc}(\frac{d\gamma }{dlogC\,})$$

where *R* is the gas constant, *T* is the temperature; *C* is the molar concentration of gemini surfactants. For gemini surfactants, *n* is taken as 3. Surface excess is an amount of feasibility of the surfactant adsorption. The values of surface excess which is the measure of bunching and stiffness of particles at the interface are important for many applications such as enhanced oil recovery, floatation, soil remediation, and detergency. It is reported in literature^49^ that on increasing the spacer chain length of gemini surfactants, the ${\Gamma }_{max}$ values increase. In the present work 16-6-16 having larger spacers has higher values than the other two. But the value for 16-5-16 is lower than 16-4-16. This abnormal characteristic of 16-5-16 may be due to different conformational arrangements at odd spacer (5) than even^50^. On increasing the spacer chain length there is the decrease in Γ_max_ values also reported in literature^51^. At the surface the values of minimum area of the per surfactant molecule (*A*_min_) by using ${\Gamma }_{max}$ values computed as

4 $${A}_{min}=\frac{{10}^{18}}{{N}_{A}{\Gamma }_{max}}$$

where *N* is Avogadro’s number. The fashion in the values of *A*_min_ is just reverse to that of ${\Gamma }_{max}$.

The cmc values of gemini surfactants also evaluated by conductometry. The cmc values were computed from the plot of specific conductivity (κ) vs. surfactant concentration (Fig. 2). The cmc values obtained from conductivity were slightly higher than obtained by surface tension measurements. The stern layer of the ionic micelle binds counterions that move migrate with micelle in the electric field. At higher concentration, ionic surfactants behave like strong electrolytes (dissociate completely) and conductance increase linearly up to cmc. After such a critical point the conductance decrease with concentration confirm the binding of some counterions to the micelles that lead to a reduction in effective charge on the micelles. The amount of bounding electrons with micelle or degree of ionization can be computed by the fractions of the pre and post-micellar slopes. The fraction of counterions bound with the micelle (surface charge density) can be calculated by the equation

5 $$g=(1-\frac{{S}_{2}}{{S}_{1}})$$

The values of g are listed in Table 1. The 16-6-16 has higher g value.

### Influence of pH variables

As the role of pH is important on the reaction, the effect of various pH was observed in geminis by fixing other experimental parameters constant, i.e., reactants and temperature. The observed rate constants (k_ψ_) values are mentioned in Table 2. It is detected that rate constants rise up to pH (5.0) thereafter become nearly invariant. It is documented well in previous reports that Schiff base development (>C=N–) is an acid-catalyzed and optimum pH is 5.0^52,53^. The product formed in the present case also consists of the same kind of linkage. Thus, whole runs were executed at pH 5.0.

**Table 2:** Effect of [Zn(II)-Gly-Leu]^+^, pH and temperature on rate constant (*k*_Ψ_) in 16-*s*-16 gemini surfactants. *Experimental conditions*: [16-*s*-16] = 30 × 10^−5^ mol dm^−3^.

10^4^ [Zn(II)-Gly-Leu]^+^ 10^3^ [Ninydrin] pH			Temp. (K)	10^5^ *k*_ψ_ (*s*-1)		
(mol dm^−3^)	(mol dm^−3^)			16-6-16	16-5-16	16-4-16
1.0	6	5.0	343	4.5	5.2	6.2
1.5				4.4	5.3	6.2
2.0				4.5	5.3	6.1
2.5				4.5	5.0	6.1
3.0				4.4	5.3	6.2
2.0	6	4.0	343	1.2	1.8	2.3
		4.5		2.2	3.5	4.2
		5.0		4.5	5.3	6.1
		5.5		4.8	5.5	6.5
		6.0		5.1	5.7	6.8
2.0	6	5.0	343	4.5	5.3	6.1
	10			7.5	9.2	10
	15			14.2	15.8	16.8
	20			20.1	21.7	23.1
	25			25.5	26.8	27.8
	30			28.6	29.6	30.5
	35			29.5	31.5	33
	40			30.2	33	35.1
2.0	6	5.0	333	1.8	2.8	3.6
			338	3.0	3.8	4.5
			343	4.5	5.3	6.1
			348	6.5	8.1	9.6
			353	9.0	10.5	11.7

### [Zn(II)-Gly-Leu]^+^ concentration

The observed rate constants were evaluated at various initial concentrations of complex keeping other reaction ingredients constant in gemini surfactant medium. In the presence of surfactants, the reaction was first order in [Zn(II)-Gly-Leu]^+^ complex as *k*_ψ_-values do not depend on initial [complex] (Table 2). Equation (6) is defined as:

6 $${\rm{d}}[{\rm{Product}}]/{\rm{dt}}={{\rm{k}}}_{\psi }[{\rm{complex}}]$$

*k*_ψ_ and [complex] represent the rate constant and total concentration of zinc-glycylleucine, respectively.

### Ninhydrin concentration

**Figure 4:**
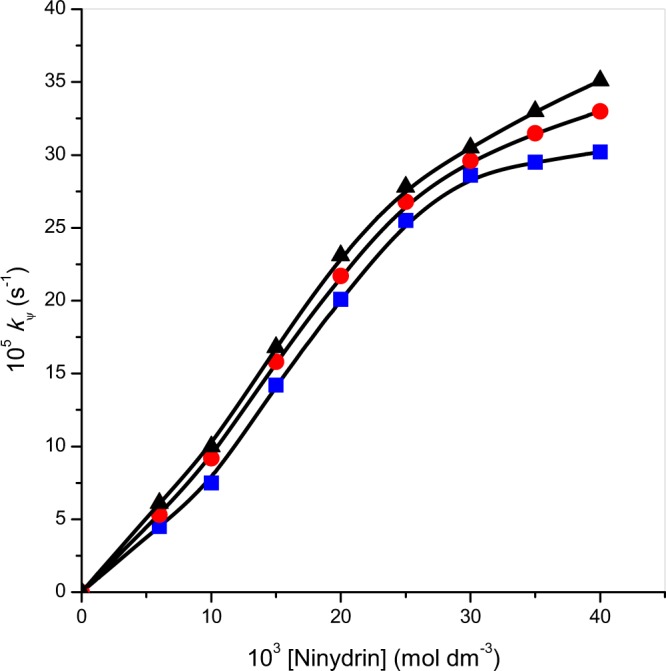
Effect of [ninhydrin] on rate of ninhydrin and [Zn(II)-Gly-Leu]^+^ reaction in 16-*s*-16 micelles: (■) 16-6-16, (●) 16-5-16, (▲) 16-4-16. *Experimental conditions*: [Zn(II)-Gly-Leu]^+^ = 2 × 10^−4^ mol dm^−3^, [16-*s*-16] = 30 × 10^−5^ mol dm^−3^, temperature = 343 K and pH = 5.0.

Effect of ninhydrin on the title reaction in 16-s-16 micellar medium was seen at [Zn(II)-Gly-Leu]^+^, temperature and pH constant. The evaluated data of rate constant at different ninhydrin concentration range is summarized in Table 2. The graph of the rate constant against ninhydrin concentration is presented in Fig. 4. The curves of Fig. 4 show the non-linear plots of kψ against [ninhydrin] and crosses through the origin, which confirms an order to be fractional in ninhydrin in the presence of 16-s-16 surfactants.

### Temperature

Studies have been made at five different temperatures varied from 333 K to 353 K with 5 K interval range. The rate constants determined at different temperatures in gemini micellar media are mentioned in Table 2. Pseudo-first-order rate constants increased on increasing temperature. Various thermodynamic parameters such as activation enthalpy, Δ*H*^#^, activation entropy, Δ*S*^#^ and activation energy, *E*_a_ were evaluated from the Eyring equation. These values of thermodynamic parameters are kept in Table 3.

**Table 3:** The values of thermodynamic parameters, rate and binding constants for condensation reaction of ninhydrin and [Zn(II)-Gly-Leu]^+^ complex at different temperatures in 16-*s*-16 gemini surfactant micelles.

Parameters	Aqueous	16-6-16	16-5-16	16-4-16
*E*_a_ (kJ mol^−1^)	62.9	47.8	45.8	43.4
Δ*H*^#^(kJ mol^−1^)	60.1	45.0	43.0	40.6
−Δ*S*^#^ (JK^−1^ mol^−1^)	130.0	142.1	142.7	143.5
10^2^ *k*_m_ (*s*-1)^a^	—	3.0	4.2	5.7
*K*_S_ (mol^−1^ dm^3^)^a^	—	78.0	74.0	69.0
*K*_N_ (mol^−1^ dm^3^)^a^	—	76.0	73.2	70.1

*Experimental conditions:* [ninhydrin] = 6 × 10^−3^ mol dm^−3^, [Zn(II)-Gly-Leu]^+^ = 2 × 10^−4^ mol dm^−3^, [16-*s*-16] = 30 × 10^−5^ mol dm^−3^ and pH = 5.0.

^a^at 298.15 K.

## Discussion

### Reaction mechanism of study

**Scheme 2:**
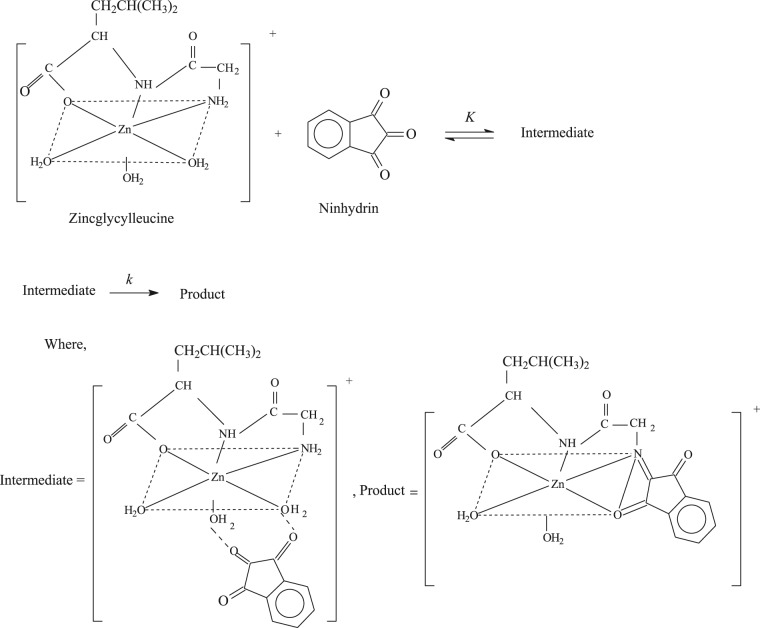
Condensation reaction between zincglycylleucine complex and ninhydrin. Where, K and k stand for equilibrium and rate constant, respectively.

In the current study, considering the cognizance of results, Scheme 2 was suggested for this reaction. It is established that lone pair electrons of an amino group of [Zn(II)-Gly-Leu]^+^ complex are required for nucleophilic attack on the carbonyl group of ninhydrin. As shown in Scheme 2, the nucleophilic attack is not probable because electrons of lone pair are not free in [Zn(II)-Gly-Leu]^+^. The condensation, therefore, continues through the carbonyl group of ninhydrin to an amino group of glycyl-leucine within the coordination sphere of the same zinc metal. This kind of interaction of reactants with the same metal ion into its coordination sphere is an existence of template mechanism^54,55^.

### Rate–[16-s-16] plots

**Figure 5:**
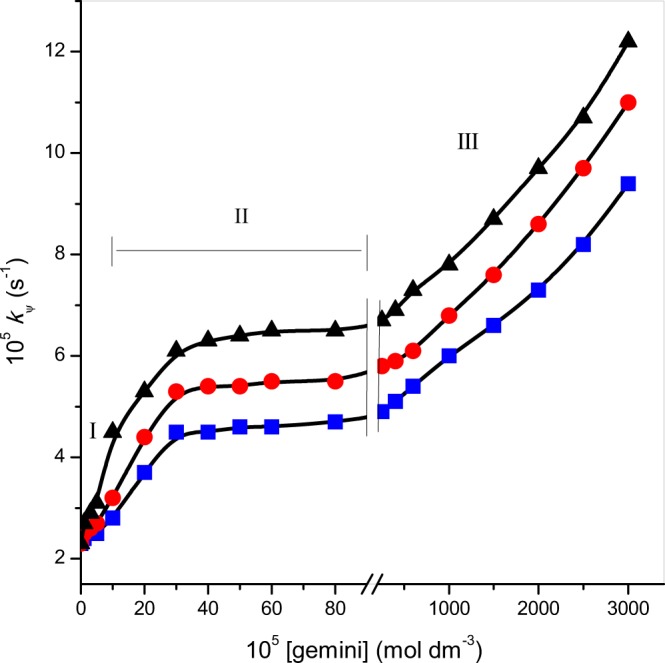
Effect of [16-s-16] gemini surfactants on rate of ninhydrin and [Zn(II)-Gly-Leu]^+^ reaction: (■) 16-6-16, (●) 16-5-16, (▲) 16-4-16. *Experimental conditions*: [ninhydrin] = 6 × 10^−3^ mol dm^−3^, [Zn(II)-Gly-Leu]^+^ = 2 × 10^−4^ mol dm^−3^, temperature = 343 K and pH = 5.0.

The role of 16-s-16 gemini surfactants on rate constants has been seen by varying amounts of [16-s-16] (0 to 3000 × 10^−5^ mol dm^−3^) while other conditions were kept constant (Table 4). Detailed experiments led to the conclusion that the same first-order path to complex and fractional-order path to [ninhydrin] was observed in gemini micellar medium as that of the aqueous medium; confirming the formation of the same product in each case. Rate vs. [16-s-16] plots were shown graphically in Fig. 5.

**Table 4:** Effect of [16-*s*-16] on condensation reaction of ninhydrin and [Zn(II)-Gly-Leu]^+^ complex and the comparison of values between *k*_ψ_ and *k*_ψcal_. *Experimental conditions:* [ninhydrin] = 6 × 10^−3^ mol dm^−3^, [Zn(II)-Gly-Leu]^+^ = 2 × 10^−4^ mol dm^−3^, temperature = 343 K and pH = 5.0.

10^5^[16-*s*-16] (mol dm^−3^)	16-6-16		16-5-16		16-4-16	
	10^5^ *k*_ψ_ (*s*-1)	10^5^ *k*_ψcal_ (*s*-1)	10^5^ *k*_ψ_ (*s*-1)	10^5^ *k*_ψcal_ (*s*-1)	10^5^ *k*_ψ_ (*s*-1)	10^5^ *k*_ψcal_ (*s*-1)
0	2.3	**—**	2.3	**—**	2.3	**—**
1.0	2.4	**—**	2.5	**—**	2.7	**—**
3.0	2.5	**—**	2.6	**—**	2.9	**—**
5.0	2.5	**—**	2.7	**—**	3.1	**—**
10.0	2.8	**—**	3.2	**—**	4.5	**—**
20.0	3.7	3.5	4.4	4.3	5.3	5.1
30.0	4.5	4.3	5.3	5.0	6.1	5.8
40.0	4.5	4.5	5.4	5.5	6.3	6.4
50.0	4.6	4.7	5.4	5.4	6.4	6.3
60.0	4.6	4.8	5.5	5.3	6.5	6.7
80.0	4.7	4.5	5.5	5.6	6.5	6.4
100.0	4.8	4.6	5.7	5.9	6.6	6.8
250.0	4.9	5.0	5.8	5.8	6.7	6.7
400.0	5.1	5.1	5.9	6.0	6.9	6.6
600.0	5.4	5.6	6.1	6.3	7.3	7.2
1000.0	6.0	**—**	6.8	**—**	7.8	**—**
1500.0	6.6	**—**	7.6	**—**	8.7	**—**
2000.0	7.3	**—**	8.6	**—**	9.7	**—**
2500.0	8.2	**—**	9.7	**—**	10.7	**—**
3000.0	9.4	**—**	11.0	**—**	12.2	**—**

**Scheme 3:**
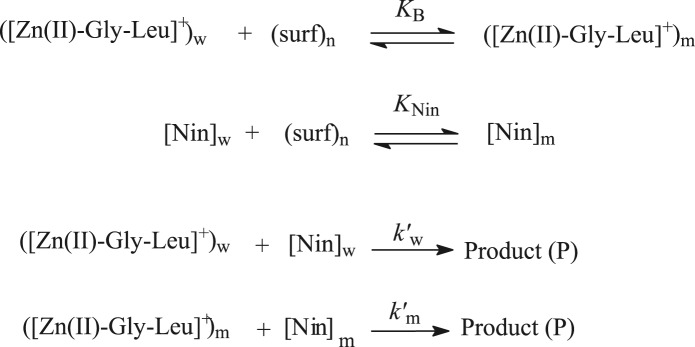
Study of ninhydrin and zinc-glycylleucine complex reaction.

In order to explain the catalytic effect of 16-s-16 gemini surfactants on ninhydrin and [Zn(II)-Gly-Leu]^+^ reaction, the observed data may be rationalized by the model (*pseudo-phase*) of surfactants suggested by Martinek *et al*.^56^ and by Menger^57^ and established by Bunton^58,59^. Under such kind of reaction situation condition, Scheme 3 can be given as below:

[Nin]_T_ and (surf)_n_ define the total concentration of ninhydrin and micellized surfactant (=[16-s-16]-cmc), respectively. w and m express respective bulk and micellar media.

Equation (6) and Scheme 3 gave Eq. (7):

7 $${k}_{\psi }=\frac{{k{\prime} }_{{\rm{W}}}+{k{\prime} }_{{\rm{m}}}{K}_{{\rm{B}}}[{({\rm{surf}})}_{{\rm{n}}}]}{1+{K}_{{\rm{B}}}{[({\rm{surf}})}_{{\rm{n}}}]}$$

Equation (7) led as Eq. (8):

8 $${k}_{\Psi }=\frac{{k}_{{\rm{w}}}{[{\rm{Nin}}]}_{{\rm{T}}}+({K}_{{\rm{B}}}{k}_{{\rm{m}}}-{k}_{{\rm{w}}}{){\rm{M}}}_{{\rm{N}}}^{{\rm{S}}}{[({\rm{surf}})}_{{\rm{n}}}]}{1+{K}_{{\rm{B}}}{[({\rm{surf}})}_{{\rm{n}}}]}$$

9 $${k}_{{\rm{w}}}=\frac{{k{\prime} }_{{\rm{W}}}}{{[({\rm{Nin}})}_{{\rm{W}}}]}$$

10 $${k}_{{\rm{m}}}=\frac{{k{\prime} }_{{\rm{m}}}}{{{\rm{M}}}_{{\rm{N}}}^{{\rm{S}}}}$$

11 $${{\rm{M}}}_{{\rm{N}}}^{{\rm{S}}}=\frac{[{({\rm{Nin}})}_{{\rm{m}}}]}{{[({\rm{surf}})}_{{\rm{n}}}]}$$

where, ${{\rm{M}}}_{{\rm{N}}}^{{\rm{S}}}$ is concentration of ninhydrin in molar ratio of the micellar head group.

The best fit values of K_B_ (binding constant for complex), K_Nin_ (binding constant for ninhydrin) and *k*_m_ (micellar rate constant) have been determined by employing a computer program and mentioned in Table 4^60^. The authenticity of rate Eq. (7) is proven by matching the observed rate constant (k_ψ_-values) and calculated rate constant (k_ψcal_-values) with a close agreement. These determined values are mentioned in Table 4.

In the current situation, rate constants, k_ψ_, increased on increasing gemini surfactants (where, [16-s-16] are below cmc values, region I) and remained almost fix (region II). Curve features of (region I and II) gemini surfactants ([gemini] = 0 to 400 × 10^−5^ mol dm^−3^) are similar as a CTAB (single hydrophilic head group and hydrophobic part). Thereafter, a sharp increment in rate was found with higher gemini surfactant concentrations, region III (Fig. 5). Critical micelle concentration (cmc) of the surfactant molecules is an essential feature that reveals its micellization capability. Physico-chemical properties surfactant molecules vary, remarkably, below and above the cmc values of surfactants^61–66^.

In region I, at [16-s-16] are lower than its cmc value, rate, k_ψ_, should be stayed fix. But, enhancement in rate was observed and this may be happened due to pre-micelles and/or preponement of micellization by substrate^67^. The present behavior was also supported by previous literature where pre-micellization and catalysis below cmc value can be found^68^.

While no reaction was occurred in region II and k_ψ_ turned about to be fixed for 16-s-16 gemini surfactants. The intent behind the consistent in k_ψ_ value can occur when the substrate is absolutely micellar bounded with micellar assembly regarded to persist unaffected^69^.

Outcomes of region III are more astonishing, i.e., rapid increment in k_ψ_ are noticed with increasing 16-s-16 concentration; probably causes a change in aggregates/morphologies of micelle.

After leveling-off, *k*_ψ_-value upturns further at higher [16-s-16]. Possibly, the reason behind this is the aggregation of surfactants in different shapes or structures. A lot of researchers proved that the surfactants self-associated to make a micelle above a certain concentration known as cmc. Normally, surfactants monomers aggregation or association gives rise to spherical shape micelle. Although, the shape may be changed from spherical to the worm-like structure after undergoing uniaxial growth at suitable physical parameters (temperature, pressure, concentration, salinity, presence of counterions, etc.)^70^. But for the gemini surfactants, the uniaxial progress and the formation of different forms of association of monomers also depend on spacer chain length. Danino *et al*.^71^, demonstrated by the Cryo-TEM study that the solution of gemini (16-s-16) on increasing the spacer chain lengths undergoes the transition from vesicles + elongated micelles → elongated micelles → spheroidal micelles. Therefore, it is concluded that the surfactant having shorter spacer length has more noticeable micellar growth as a result of the more geometrical constraints in the creation of micelle.

The change in aggregates/morphologies also confirmed by inspecting of ^1^H NMR studies of geminis by Brinchi *et al*.^72^. Therefore, at higher concentration, k_ψ_ rises up due to change in aggregate morphologies of 16-s-16 and lead diverse environment (less polar) (Fig. 5).

### Thermodynamic parameters

**Chart 1:**
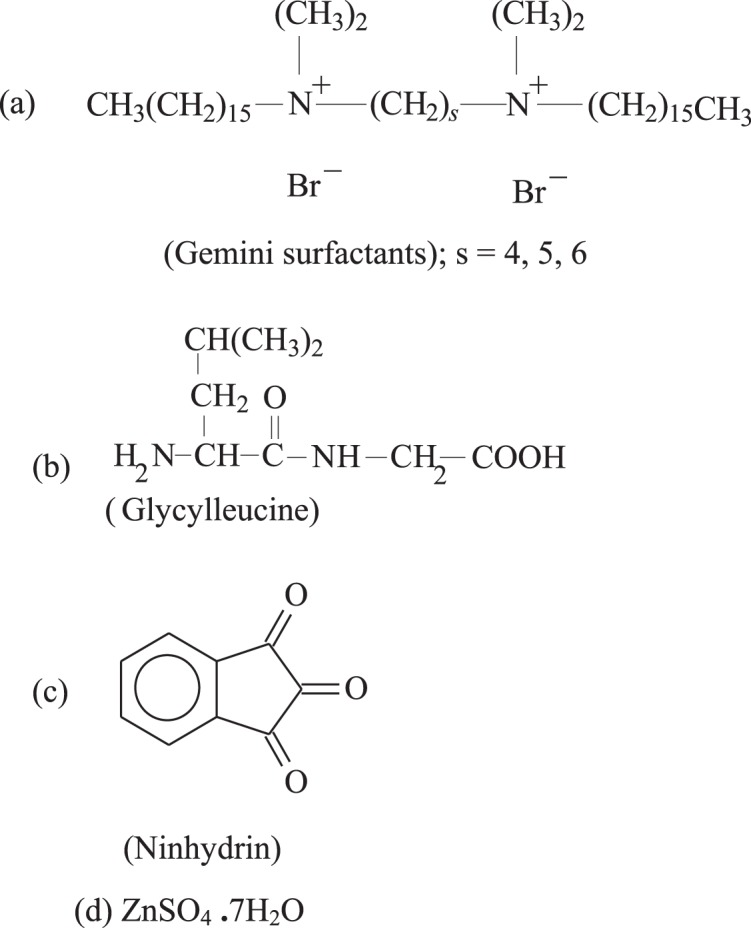
Molecular structure of gemini surfactants and reactants used in the current study.

Several parameters, e.g., activation enthalpy, Δ*H*^#^, activation entropy, Δ*S*^#^ and activation energy, *E*_a_ were calculated on the study of ninhydrin and zinc-glycylleucine complex using Eyring equation. Table 3 consists of the values of thermodynamic parameters. The catalysis of 16-s-16 geminis through the interaction of ninhydrin and zinc-glycylleucine is disclosed by values of Δ*H*^#^ and Δ*S*^#^. The reduced value of enthalpy of activation occurs through adsorption of the substrate on micellar surface and stabilization of transition state in geminis as compared to aqueous^73^. Low Δ*S*^#^ value (with a substantial negative entropy) indicates that a well-structured activated complex is formed in gemini micelles than aqueous medium. The decrease in activation energy, *E*_a_, leads that the 16-s-16 surfactants function as a catalytic agent and provide a new idea for the reaction. A significant mechanistic explanation of these apparent parameters is not possible because k_ψ_ does not signify a single elementary kinetic step; it is a complex function of true rate, binding and ionization constants (Chart 1).

## Conclusions

The study deals with the synthesis and characterization of gemini surfactants and their influence on the kinetic behavior of ninhydrin with zinc-glycylleucine. The reason for choosing this particular study is the mechanism of ninhydrin with amino acids reaction in water, conventional surfactants (CTAB, cetylpyridinium bromide, CPB) and the various solvent medium is well recognized^24–30,59^. Under the identical reaction condition, it was found that the study was catalyzed and accelerated quite enough in gemini surfactants (below their cmc value) than aqueous medium^73^. This confirms that a smaller quantity of synthesizing reagents is necessary for the synthesis of gemini surfactants. The above leads to the conclusion that consumption of the little amount of surfactant offers a less impact on environmental toxicity as well as cost-effectiveness.

At the present case, a lower value of k_ψ_ and K_B_ are observed in gemini surfactants than [Ni(II)-Gly-Phe]^+^ ^37^. A plausible explanation difference between in k_ψ_ and K_B_ could be related to the fact that glycylphenylalanine is more hydrophobic than glycylleucine. Enhanced hydrophobicity seems responsible for a higher concentration of glycylphenylalanine in the Stern layer of gemini surfactants.
